# Imaging of tumor clones with differential liver colonization

**DOI:** 10.1038/srep10946

**Published:** 2015-06-22

**Authors:** Go Oshima, Sean C. Wightman, Abhineet Uppal, Melinda E. Stack, Sean P. Pitroda, Jonathan J. Oskvarek, Xiaona Huang, Mitchell C. Posner, Samuel Hellman, Ralph R. Weichselbaum, Nikolai N. Khodarev

**Affiliations:** 1 Department of Surgery; 2 Department of Radiation and Cellular Oncology, Ludwig Center for Metastasis Research, The University of Chicago, Chicago, IL 60637.

## Abstract

We present a model of hepatic colorectal metastases which represents monoclonal cell lines double-labeled by luciferase and tdTomato. These cells form liver metastasis in varying numbers and patterns similar to those observed in patients. Using *in vivo* and *ex vivo* luminescent and fluorescent imaging we determine the growth kinetics and clonogenic frequency of tumor cells colonizing liver. Molecular profiling detected stable expressional differences between clones consistent with their phenotypes. The data indicate that clinically relevant phenotypes of liver metastases can be modeled *in vivo*.

Patients with colorectal cancer often present with liver metastases, which frequently results in a fatal outcome. Between 5 to 20% of patients with limited numbers of hepatic metastases and slow rates of progression/recurrence are successfully treated with local treatment approaches, with or without systemic therapy^1^,^2^. Little is known about liver metastases heterogeneity and animal models that reflect heterogeneity are still absent.

The tumor burden of hepatic metastases has been previously quantified by weight or volume of whole liver or the macroscopic findings of liver tumors^3^,^4^,^5^. Alternatively, models of experimental liver metastasis based on luminescently-labeled tumor cells have been previously reported^6^,^7^. However there are no reports about selection of tumor clones containing double luminescent and fluorescence tags and demonstrating different abilities to colonize and grow in the liver microenvironment. Successful development of such model can significantly assist in the understanding of mechanisms of development of liver metastases.

Here we present a new approach to model liver metastases, which includes generation of tumor clones with different liver colonization efficiencies and growth properties. This model represents monoclonal populations of tumor cells recapitulating oligometastatic, potentially curable disease and widespread metastatic disease. We employ dual labeling of clones with luciferase and tdTomato to provide different *ex vivo* and *in vivo* imaging modalities, including diffuse luminescent Imaging tomography (DLIT) for detection and resolution of individual tumor colonies during the time course of liver colonization. Our data indicate that observed phenotypes of liver metastases are based on the combination of two parameters- the efficiency of tumor colonization and doubling time (Td) following adaptation of tumor cells to the liver microenvironment.

## Results

### Generation of a panel of monoclonal cell lines with different metastatic ability

**Figure 1:**
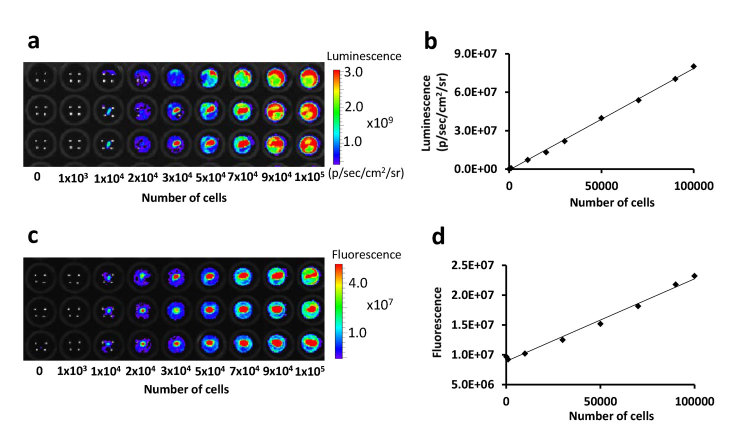
The correlation between the number of cells and luminescence or fluorescence in vitro. (**a**) The luminescent image of different number of cells triplicated in 96-well plate. (**b**) The correlation between the number of cells and luminescence (R = 0.99, p < 0.0001). (**c**) The fluorescent image of different number of cells triplicated in 96-well plate. Intensities were acquired as radiant efficiency with 535 nm excitation and 580 nm emission. (**d**) The correlation between the number of cells and fluorescence (R = 0.99, p < 0.0001).

To generate a panel of labeled monoclonal HCT116 cell lines, HCT116 cell lines were double-labeled with Luc2 and tdTomato proteins^8^. tdTomato-positive cells were collected using flow cytometry and 16 monoclonal double-labeled HCT116 cell lines (HCT116-L2T) were generated. In vitro fluorescence and luminescence of HCT116-L2T were quantified using increasing amounts of cells, and both fluorescence and luminescence were in good correlation with cell numbers (Pearson’s R = 0.99; *p* < 0.0001) (Fig. 1).

**Figure 2:**
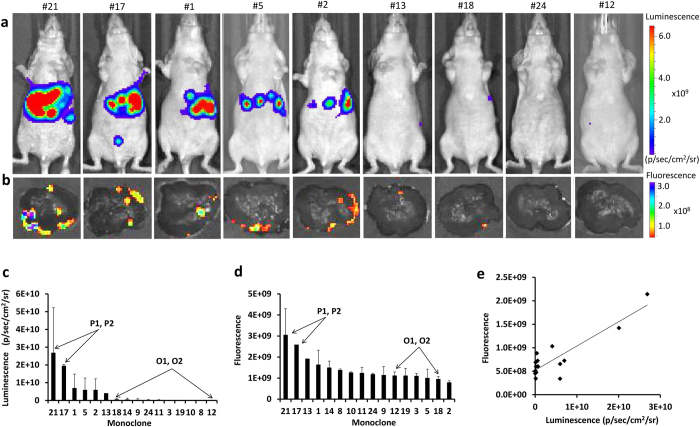
Bioluminescence and *ex vivo* fluorescence of liver in 16 clones at 2 weeks after spleen injection. (**a**) Representative bioluminescent images. (**b**) Representative *ex vivo* fluorescent images of harvested livers. Intensities were acquired as radiant efficiency with 535 nm excitation and 580 nm emission. (**c**) The distribution of bioluminescent intensities of each clone. (**d**) The distribution of *ex vivo* fluorescent intensities of each clone. (**e**) The correlation between *in vivo* bioluminescence and *ex vivo* fluorescence (R = 0.89, *p* < 0.0001).

Sixteen individual clones were injected into spleen followed by splenectomy (Supplementary Video 1, 2, 3, 4, 5). Bioluminescence of mice and *ex vivo* fluorescence of harvested livers were quantified at 2 weeks after spleen injection (Fig. 2a,b). Quantification of these data demonstrated that clones had different abilities to colonize and grow in the liver as was determined by both luminescent measurements *in vivo* and fluorescent *ex vivo* imaging (see Fig. 2c,d). Clones #21 and #17 which showed high propensity to colonize liver had overall luminescence equal to 2.7 × 10^10^ ± 2.5 × 10^10^ and 2.0 × 10^10^ ± 7.7 × 10^8^ p/sec/cm^2^/sr (steradian). Clones #12 and #18 with low colonization had overall luminescence equal to 2.4 × 10^7^ ± 3.0 × 10^7^ and 6.6 × 10^8^ ± 4.1 × 10^8^ p/sec/cm^2^/sr, respectively. We correlated *in vivo* luminescence and *ex vivo* fluorescence for each clone (Pearson’s R = 0.89; *p* < 0.0001) (Fig. 2e). We further selected clones #12 and #18 as “oligometastatic” clones, potentially recapitulating limited metastatic disease and designated them as O1 and O2, respectively. We also selected clones #21 and #17 as further model for widespread dissemination, also referred to as polymetastatic disease, in liver and named them P1 and P2, respectively (Fig. 2c,d).

**Figure 3:**
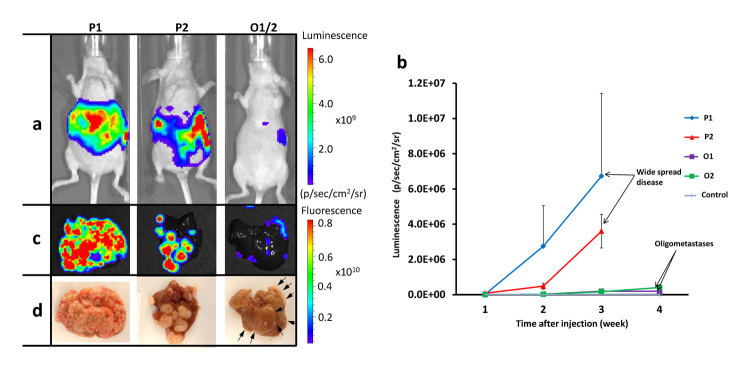
Bioluminescence and *ex vivo* fluorescence of liver in selected clones P1, P2, O1 and O2. (**a**) Representative bioluminescent images, (**b**) Bioluminescence weekly measured from 1 to 3 weeks in P1 and P2, 1 to 4 weeks in O1 and O2. (**c**) Representative *ex vivo* fluorescent images of livers. Intensities were acquired as radiant efficiency with 535 nm excitation and 580 nm emission. (**d**) Macroscopic liver images (arrows indicate white small tumors). P1 and P2; polymetastatic clones, O1 and O2; oligometastatic clones.

To further characterize these metastatic phenotypes clones O1, O2, P1 and P2 were intrasplenically injected in corresponding groups of mice and bioluminescence was measured weekly. *Ex vivo* fluorescence of livers were quantified at 4 weeks after spleen injection. The bioluminescence was higher in P1 and P2 mice (6.7 × 10^6^ ± 4.7 × 10^6^ and 3.6 × 10^6^ ± 2.5 × 10^6^ p/sec/cm^2^/sr) than in O1 and O2 mice (2.0 × 10^5^ ± 1.3 × 10^5^ and 1.7 × 10^5^ ± 9.1 × 10^4^ p/sec/cm^2^/sr) 3 weeks after spleen injection (Fig. 3a,b). The *ex vivo* fluorescence in P1 and P2 was higher than in O1 and O2; this was consistent with macroscopic findings (Fig. 3c,d).

### Quantification of colonization ability and growth properties of selected clones

**Figure 4:**
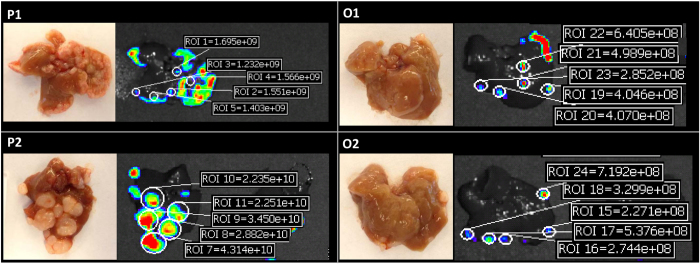
Quantification of fluorescent intensities of individual liver colonies. Left images indicate representative macroscopic findings and right images indicate fluorescent intensities in each clone. The fluorescent intensities were acquired as radiant efficiency with 535 nm excitation and 580 nm emission. P1 and P2; polymetastatic clones, O1 and O2; oligometastatic clones.

**Figure 5:**
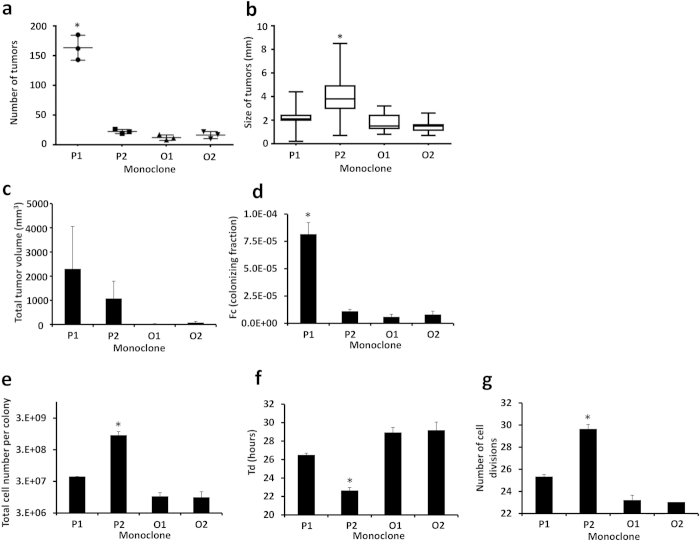
Quantitative estimation of colonizing ability and growth kinetics. (**a**) The macroscopic total number of tumors, (**b**) macroscopic size of individual tumors, (**c**) calculated total tumor volume in liver, (**d**) Fc; colonizing fraction of tumor colony forming cells, (**e**) total cell number per colony, (**f**) doubling time and (**g**) number of cell divisions of each clone. P1 and P2; polymetastatic clones, O1 and O2; oligometastatic clones.

For further evaluation of metastatic colonization we measured macroscopically defined numbers and sizes of tumors and *ex vivo* fluorescence of individual colonies (Fig. 4). The total number of tumors was higher in P1 than P2, O1 and O2, whereas the size of tumors in P2 was larger than in P1, O1 and O2 (p < 0.001) (Fig. 5a,b). However total tumor volume in liver was larger in P1 and P2 than O1 and O2 (Fig. 5c). The colonizing ability of the tumor cells is expressed as the colonizing fraction (Fc) which is calculated as the number of tumors in liver divided by the number of cells injected. Assuming that the number of tumors in liver corresponds to the number of cells which have an ability to colonize liver, P1 had more ability to colonize liver than the other clones (Fc = 8.2 × 10^−5^ ± 1.1 × 10^−5^, 6.0 × 10^−6^ ± 2.3 × 10^−6^ in P1 and O1, respectively, *p* < 0.001) (Fig. 5d). P2 had similar ability to colonize the liver as clones O1 and O2, but the size of tumor was larger than the other clones. We further correlated total tumor volumes and *in vivo* luminescence or *ex vivo* fluorescence for each investigated clone. As is shown in Supplementary Fig. S1, these parameters were in good correlation with each other (Pearson’s R = 0.99; *p* < 0.0001).

To characterize the basic properties of tumor colony forming cells, we calculated the total cell number per colony, doubling time (Td) and number of cell divisions by the calibration from correlation between fluorescent intensity and number of cells (Fig. 1 and Fig. 5e–g). P1 which had many small tumors showed larger colonizing fraction (Fc) and Td similar to O1 and O2 (*p* < 0.05). P2 which had large tumors showed similar Fc but shorter Td than O1 and O2 (*p* < 0.05). The data indicated that there are two parameters which define the characteristics of metastatic potential- colonizing fraction (Fc) and doubling time (Td) of tumor colony forming cells. Consistent with *in vivo* data, P2 showed faster growth as compared with the other clones *in vitro* (Supplementary Fig. S2).

**Figure 6:**
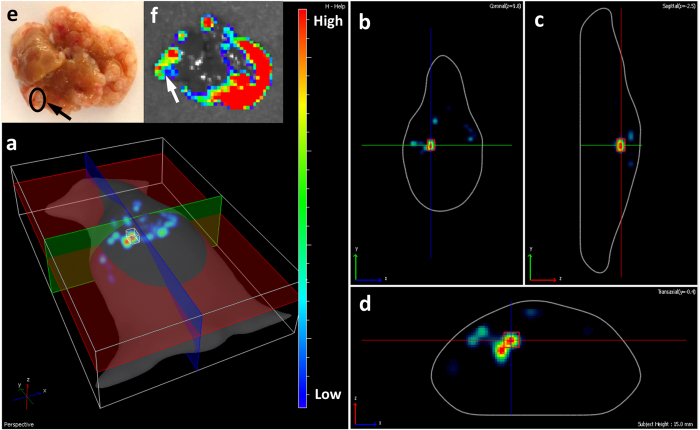
Representative images of quantification of bioluminescence of individual tumor using diffuse luminescent imaging tomography (DLIT) in P1- polymetastatic clone. (**a**) 3 dimensional overhead view, (**b**) coronal section, (**c**) sagittal section, (**d**) transaxial section, (**e**) macroscopic image of liver, (**f**) *ex vivo* fluorescent image of liver.

Diffuse luminescent Imaging tomography (DLIT) provided the 3D distribution of metastatic tumors in liver (Supplementary Video 6). Using this technique it was possible to detect and measure the bioluminescence of individual hepatic metastatic colonies in the time course of metastases development therefore tracing spatial-temporal dynamic behavior of metastases (Fig. 6).

### Molecular profiling of selected monoclones

**Figure 7:**
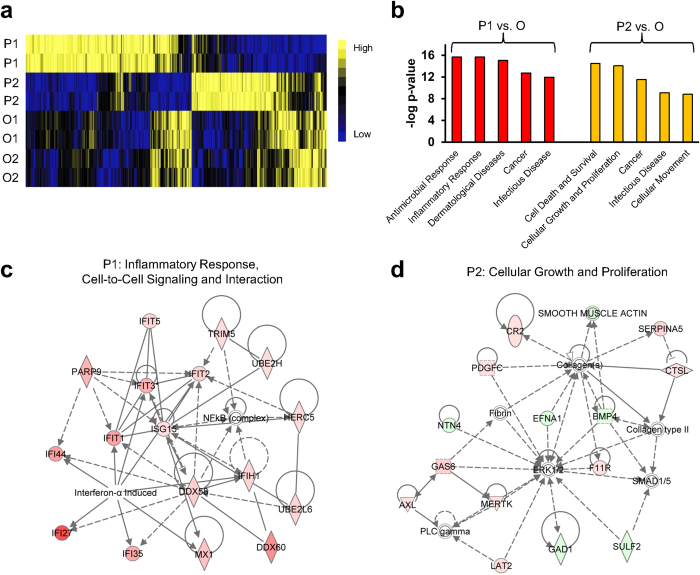
Gene expression differences between polymetastatic and oligometastatic clones. (**a**) Hierarchical clustering of 1,225 genes differentially expressed (fold-change ≥1.5 and FDR ≤5%) in P1 and P2 as compared to O clones. (**b**) Functional annotation of differentially expressed genes in P1 (left) and P2 (right) vs. O clones. Shown are the top 5 functions for each comparison. Values represent the -log10 p-value. (**c**, **d**) Ingenuity Network analysis of P1 (**c**) and P2 (**d**) specific gene patterns. Red color indicates over-expression and green color indicates suppression. Solid line indicates activation; dashed line indicates deactivation. P1 and P2; polymetastatic clones, O clones; O1 and O2, oligometastatic clones.

To detect the molecular differences between poly- and oligometastatic clones we used expressional profiling of corresponding cell lines in vitro with Illumina HT 12v4 bead arrays. We found 756 differentially expressed genes (DEGs) in P1 as compared to O1 and O2 and 461 differentially expressed genes in P2 as compared to O1 and O2 (Fig. 7a and Supplementary Table S1, S2). To estimate the functional significance of the detected DEGs we used Ingenuity Pathway Analysis (IPA) as previously described before^9^,^10^,^11^. As is shown in Fig. 7b, P1 is enriched by constitutively expressed genes associated with inflammatory responses. Interestingly, many of these genes are presented by an interferon-related gene signature (IRDS) previously described by us and others in the context of tumorigenicity, metastases development and radio-/chemo-resistance (Fig. 7b,c)^9^,^12^,^13^,^14^,^15^,^16^,^17^. P2 was enriched by genes, associated with regulation of growth and survival – a regulatory network of a subset of these genes is presented in Fig. 7d. Taken together, these data provide evidence that polymetastatic clones demonstrate molecular differences from oligometastatic clones. As well, two polymetastatic but phenotypically distinct clones (P1 and P2) express regulatory networks of genes which are consistent with their phenotypic properties.

## Discussion

**Figure 8:**
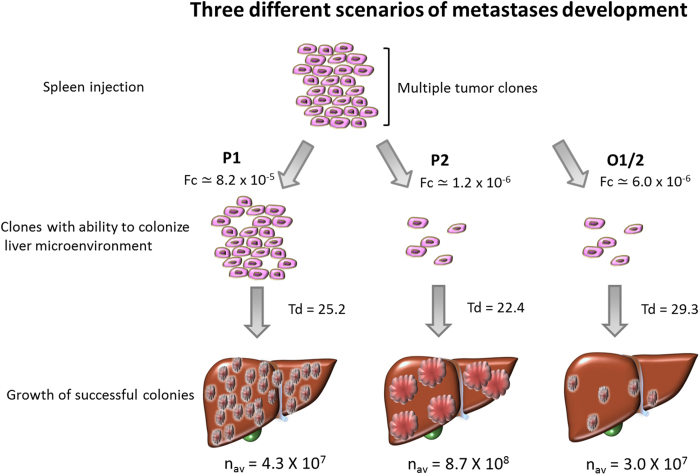
Three different scenarios of metastases development. First, P1 had higher Fc, longer Td and lower n_av_ resulted in increased number of small tumors. Second, P2 had lower Fc, shorter Td and higher n_av_ resulted in limited number of larger tumors. Thirdly, O1 and O2 had lower Fc, longer Td and lower n_av_ resulted in less number of small tumors. P1 and P2; polymetastatic clones, O1 and O2; oligometastatic clones. Fc; colonizing fraction of tumor forming cells, Td; doubling time (hours), n_av_; average number of cells per colony.

Our data indicate at least three potential scenarios of colorectal metastases development in liver which can be recapitulated in our experimental system. The first scenario presented by clone P1 is determined by high ability of the clone to colonize liver (Fc = 8.2 × 10^−5^ ± 1.1 × 10^−5^) but is associated with relatively low growth rate (Td = 26.5 ± 0.2 hours). A combination of these parameters leads to increased number of small metastatic tumors. A second case is P2, which had lower Fc (1.1 × 10^−5^ ± 1.8 × 10^−5^) and shorter Td (22.7 ± 0.3 hours) resulted in relatively small number of large secondary tumors. And a third case is presented by clones O1 and O2, which had lower Fc and longer Td resulting in less number of small tumors, which might be clinically considered as oligometastatic liver disease (Fig. 8)^18^,^19^. Indeed, clinical observations are consistent with these models. Fong and co-authors provided a comprehensive review of more than 1000 cases of hepatic resections of colorectal metastases to liver^1^. Recently it was updated to include 1600 patients with maximal follow-up time 17.4 years^20^. In both reviews, based on comprehensive statistical analysis, authors refer to the size of liver metastases (parameter related to growth potential) and their numbers (parameter related to colonization ability) as to independent risk factors for distant metastasis, which is consistent with P2 and P1 properties. Our recent observations of patients, treated by stereotactic body radiotherapy (SBRT) or with surgically resected lung metastases points both to the number and rate of progression as key factors determining overall and disease-free outcome^18^,^19^. While these findings are an oversimplification of the problem, the complexity of the metastatic processes is far from a comprehensive description^21^,^22^,^23^. However, colonizing and growth abilities seem to be among the most critical factors determining the development of metastatic clones in distant sites^24^,^25^. Experimental models, which can capture differences of metastatic clones in these abilities, may provide useful tools both for detection of molecular properties discriminating oligo- and polymetastatic pathways (Fig. 7) and for validation of potential regulatory molecules identified in high-throughput clinical screenings^11^,^18^.

An increasing number of studies have demonstrated the optical imaging as a modality for preclinical *in vivo* examination of tumor detection, growth, and response to different treatments^26^,^27^,^28^,^29^,^30^. Because of its efficiency, the clinical potential for the *in vivo* optical imaging is becoming apparent^31^,^32^. Compared to the previously reported models using cells single-labeled with luciferase^6^,^7^, our model demonstrates the advanced evaluation of tumor characterization using *ex vivo* optical imaging with fluorescence. Higher sensitivity of the bioluminescence provides the detection and quantification of small burden of metastases, and enables quantitative longitudinal tumor growth without sacrifice of the mice. However, *ex vivo* fluorescent optical imaging enables more detailed quantification of tumor distribution, has higher resolution and allows visualizing interaction of the tumor cells with surrounding tissues. In addition, it provides a unique opportunity to quantify growth parameters of metastatic colonies on the cellular level without interference of stromal tissues and has the potential to quantitatively separate tumor cells from stromal cells for their separate investigation.

In summary, we describe a new technique to model limited and widespread liver metastatic disease and to quantify the tumor burden of hepatic metastases. This technique can also provide 3D imaging of metastases distribution in liver. With quantitative estimation of tumor burden, this technique may be helpful in further investigations of the biological mechanisms of hepatic tumors and potential approaches to their treatments.

### Study approval

All studies performed on mice were approved by the IACUC of the University of Chicago.

## Methods

### Generation of clonal double-labeled HCT 116 cell line

The stably double-labeled HCT116 cell lines were generated with Luc2 and tdTomato genes using lentiviral-based gene delivery^8^. The Luc2-tdTomato plasmid and HCT116 cell line were obtained from Dr. Geoffrey Greene at the University of Chicago. The cell lines were maintained in DMEM (Life Technologies Corporation, NY, USA) with 10% fetal bovine serum,100 U/mL penicillin and 100 mg/mL streptomycin. All cell lines were maintained in culture with 5% CO_2_ at 37 degrees Celsius. The tdTomato-positive cells were collected using FACS Calibur Flow Cytometer (BD Immunocytometry Systems, CA, USA). Collected tdTomato-positive cells were diluted to 1 cell per 200 μl and plated at 1 cell per a well in 96-well plate. We generated 16 clones of HCT116 cell lines double-labeled with Luc2 and tdTomato (HCT116-L2T).

### *In vitro* quantification of fluorescent and bioluminescent signals

The HCT116-L2T cells were plated at a density of 0, 10^3^, 10^4^, 2 × 10^4^, 3 × 10^4^, 5 × 10^4^, 7 × 10^4^, 9 × 10^4^ and 10^5^ cells per well in 96-well plates. Triplicates were performed in each density of cells. After 5 hours incubation, fluorescent and luminescent intensities were quantified with IVIS 200 (Xenogen, MA, USA) imaging system. Firefly D-luciferin potassium salt (GoldBio Technology, MO, USA, 150 μg/mL per well) was added just before luminescent assay

### An animal model of hepatic colorectal metastases

All animal procedures were carried out in accordance with the approved guidelines. The procedures were approved by the Institutional Animal Care and Use Committee of the University of Chicago (Protocol # 72213-09).

Mice were anesthetized with 2% isoflurane in oxygen. Spleen was exposed through a 8 mm left flank incision (Supplementary Video 1). Six - eight weeks old female athymic nude mice (Harlan, WI, USA) were splenically injected with 2.0 × 10^6^ cells per 100 μL phosphate buffered saline into liver (n = 3, in each group) (Supplementary Video 2). Five minutes post-injection, splenectomy was performed to avoid carcinomatous peritonitis and residual growth in spleen (Supplementary Video 3). Bioluminescent intensities were measured weekly using the IVIS 200 (Xenogen, MA, USA) imaging system after intra-peritoneal injection of 150 μg firefly D-luciferin potassium salt (GoldBio Technology, MO, USA). Data were analyzed using LivingImage 4.0 Software (Caliper Life Sciences, MA, USA). Two or 4 weeks after injections, livers were harvested and *ex vivo* fluorescent intensities of liver tumors were quantified as radiant efficiency. Sixteen clones were tested to select candidates for oligo- and polymetastatic phenotypes. Diffuse luminescent imaging tomography (DLIT) was performed for evaluation of tumor burden and distribution using real-time 3D reconstruction of bioluminescent signals which provide bioluminescent intensities of individual tumor.

### Quantitative estimation of colonizing ability and growth kinetics

The numbers and sizes of liver tumors were macroscopically measured. The total tumor volumes were calculated by assuming it to be a sphere. The fluorescent intensities of 5 representative colonies in each liver were quantified using the IVIS 200 (Xenogen, MA, USA) imaging system. The fraction of tumor colony forming cells (Fc) was calculated as the number of tumors in liver divided by the number of cells splenically injected. The total cell number per colony, doubling time (Td) and number of cell divisions were calibrated from the correlation between fluorescent intensity and number of cells in vitro.

### *In vitro* growth curve

HCT116-L2T clones were plated at a density of 3,000 cells per well in 96-well plates. Triplicates were performed per cell lines. Cell growth was evaluated at 0, 24, 48, 72 and 96 hours after plating. Cells were incubated with CellTiter-Blue (Promega, WI, USA) for 3 hours and fluorescent intensities were quantified per the manufacture’s instructions. Signals were normalized to the intensities at time zero.

### Gene expression profiling and analysis

Selected HCT116 L2T clones (P1, P2, O1 and O2) were collected in cell lysis buffer, and RNA was isolated using the TRIzol Reagent (Ambion, Austin, TX). 100 ng of RNA was labeled per manufacturer’s instructions and profiled in duplicate using the Illumina Human HT12v4 array (Illumina, San Diego CA). Background subtraction and quantile normalization was performed across arrays using Illumina Beadstudio software. Log-transformed gene expression was compared using Significance Analysis of Microarrays (SAM) for Excel (Stanford University, CA) with a False Discovery Rate (FDR) of 5% and a fold-change threshold of greater than or equal to 1.5 to identify differentially expressed genes^33^.

Ingenuity Pathway Analysis (IPA, Redwood City CA) was used to identify over-represented functions and pathways. Over-representation of gene sets in a canonical pathway was calculated using hypergeometric testing with an alpha value of 0.05. Significantly enriched pathways were manually distributed into specific functional groups.

### Statistics

Data were analyzed using JMP 10 software (SAS Institute, NC, USA). Data were represented as the mean ± standard deviation for all figure panels in which error bars were shown. Pearson’s product-moment correlation coefficients were used to assess associations between parameters. The *p* values were assessed using 2-tailed Student t tests and *p* < 0.05 was considered statistically significant.

## Additional Information

**How to cite this article**: Oshima, G. *et al*. Imaging of tumor clones with differential liver colonization. *Sci. Rep*. **5**, 10946; doi: 10.1038/srep10946 (2015).

## Supplementary Material

Supplementary Information

Supplementary Video 1

Supplementary Video 2

Supplementary Video 3

Supplementary Video 4

Supplementary Video 5

Supplementary Video 6
