# Supplementary material for: Imaging of tumor clones with differential liver colonization
Source: Sci Rep. 2015 Jun 22;5:10946. doi: 10.1038/srep10946 (PMC4476146; doi:10.1038/srep10946)
Supplement: Supplementary Information [file srep10946-s1.pdf]

Supplementary Information

Imaging of tumor clones with differential liver colonization

Go Oshima[1, 2], Sean C. Wightman[1], Abhineet Uppal[1], Melinda Stack[1], Sean P. Pitroda[2], Jonathan Oskvarek[2], Xiaona Huang[2], Mitchell C. Posner[1], Samuel Hellman[2], Ralph R. Weichselbaum[2], Nikolai N. Khodarev[2]

[1]Department of Surgery; [2]Department of Radiation and Cellular Oncology, Ludwig Center for Metastasis Research, The University of Chicago, Chicago, IL 60637

Table of Contents

- Supplementary Figures S1, 2 (p. 2, 3)
- Supplementary Videos 1-6 (p. 4)
- Supplementary Table S1 (p. 5-26)
- Supplementary Table S2 (p.27-40)

Supplementary Figure S1

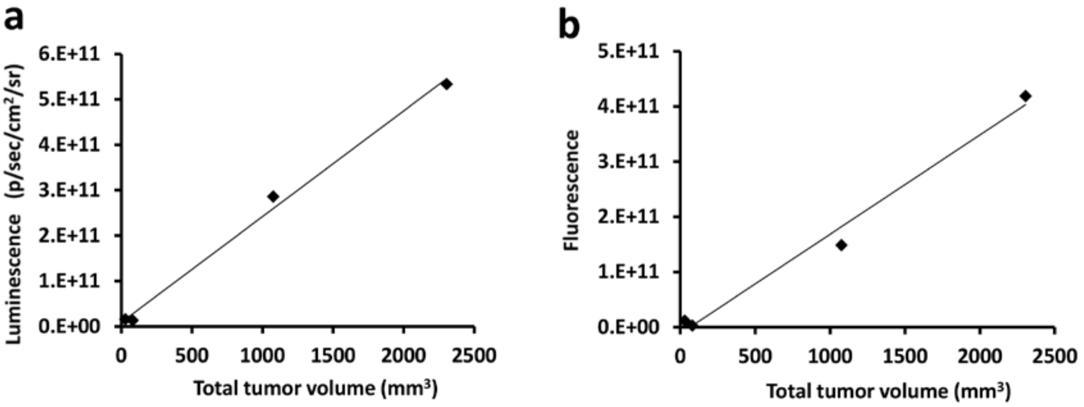

Fig. S1: The correlations between total tumor volume and bioluminescence or ex vivo fluorescence in P1, P2, O1 and O2. (a) Correlation between total tumor volume and bioluminescence (R = 0.99 , p < 0.0001). (b) Correlation between total tumor volume and ex vivo fluorescent radiant efficiency (R = 0.99 , p < 0.0001).

Supplementary Figure S2

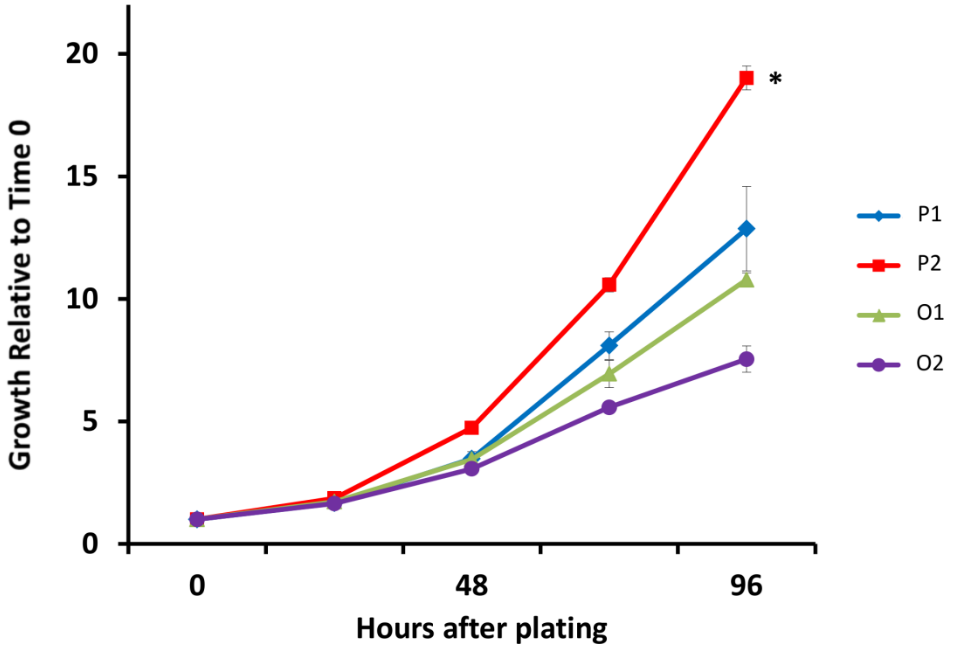

Fig. S2: Growth curve in vitro in P1, P2, O1 and O2. P1 and P2; polymetastatic clones, O1 and O2; oligometastatic clones. (*$p < 0.05$ compared with P1, O1 and O2)

Supplementary Videos 1-5: The procedure of spleen injection. (1) Step 1, opening and spleen exposure; spleen can be seen through skin as a dark area. Spleen is exposed through the 8 mm flank incision. (2) Step 2, spleen injection; the white spot is found in spleen after the injection. Hemoclip is used to prevent bleeding and dissemination of tumor cells after injection. (3) Step 3, splenectomy; the white spot disappears 5 minutes after injection. Splenectomy is performed with cautery. (4) Step 4, closure; one horizontal mattress suture is enough for wound closure. (5) Recovery from anesthesia at 1 hour after surgery.

Supplementary Video 6: DLIT of liver tumors; 3D video of diffuse luminescent imaging tomography (DLIT) in P1- polymetastatic clone.

Supplementary Table S1

Gene Symbol	Fold-Change (P1 vs. O)
MYL2	15.39
IFITM1	12.86
LOC100129681	12.74
BST2	11.52
PRR15L	10.73
ANXA9	10.22
DKK1	9.80
IFI27	9.17
PARP10	7.89
CT45A4	7.81
PLAC8	7.43
SAA1	6.74
CYP4F11	6.47
TGM2	5.89
DDX60	5.76
SUSD2	5.74
CLIC5	5.70
LDHD	5.27
FGF21	5.20
C17ORF55	5.20
RCAN1	5.08
IFI44	4.99
OAS1	4.84
IFIT1	4.74
LTK	4.72
SLC7A7	4.63
SP6	4.52
IFI35	4.44
PSD2	4.32
TRIM22	4.31
GPR162	4.25
CITED4	4.23

CHST4	4.16
RARRES3	4.16
SERPINA1	4.12
EPSTI1	4.10
IFI6	4.09
LOC400879	4.03
TNNC1	3.95
DDR2	3.92
OAS3	3.86
EBI3	3.85
ALPPL2	3.82
ATP1B2	3.81
LOC642477	3.74
SLC1A3	3.71
TLR3	3.65
CD74	3.58
SCARNA8	3.48
IFIT3	3.40
PARP9	3.40
APOC1	3.39
NCF4	3.33
ODZ3	3.30
SCARNA16	3.30
SCARNA11	3.27
ACBD4	3.24
MX1	3.20
SNORA11B	3.18
CCL5	3.17
BMP6	3.13
MTL5	3.06
KIAA1199	3.03
PAPSS2	3.03
HERC6	3.02
SP110	3.00
SCARNA14	3.00

SNORA79	2.99
FAM63A	2.98
SNCA	2.97
STAG3	2.97
SH3PXD2A	2.95
KLK5	2.95
HCP5	2.94
NFKBIL2	2.92
MUC13	2.91
RASIP1	2.90
PROM2	2.88
CCL26	2.87
OASL	2.86
IRF7	2.78
HRASLS3	2.71
DMXL2	2.71
C19ORF4	2.70
LOC730415	2.70
HLA-DRB4	2.67
FAR2	2.67
FLJ36070	2.67
PIK3AP1	2.65
GALNT9	2.63
SCARA3	2.62
NUPR1	2.61
DDX60L	2.60
GAS7	2.60
DPP4	2.60
LGALS9	2.55
LOC649970	2.55
DNAH3	2.54
DDX58	2.48
USP18	2.48
MIR614	2.47
PLSCR4	2.46

PLCE1	2.46
SCARNA12	2.45
STAT1	2.44
SULT2B1	2.44
ABHD14B	2.42
MYOM1	2.41
SP100	2.40
MAP3K12	2.39
IL15	2.38
P8	2.38
CRABP2	2.37
LOC440160	2.36
PRIC285	2.36
FLJ39632	2.35
VWA5A	2.34
CD47	2.34
FA2H	2.33
PLSCR1	2.32
IFITM3	2.31
IFIT2	2.31
C1QL4	2.31
HS.133261	2.31
CD55	2.31
ACOX1	2.29
DIP2C	2.28
PLEKHA4	2.28
GEM	2.27
FHL1	2.27
FLJ35767	2.25
LOC100133583	2.25
USP30	2.24
CLGN	2.24
LPIN2	2.24
MEF2C	2.23
ZNF280A	2.22

IFITM2	2.21
INSL5	2.21
PKP4	2.21
LOC392437	2.20
PLCXD1	2.20
RAET1L	2.20
PRKCQ	2.19
HSH2D	2.19
LOC554223	2.17
STIM1	2.17
TMEM59L	2.17
HLA-B	2.17
HLA-F	2.16
SGPP2	2.16
IFIT5	2.15
SERPINA3	2.14
LOC100128274	2.14
KRT18P28	2.14
SRGAP3	2.13
CYP2S1	2.12
IFIH1	2.11
UBE2L6	2.11
SLC27A6	2.11
MFI2	2.11
SNORA63	2.10
ABCG1	2.09
TAPBP	2.09
IGFBP2	2.08
SNORD13	2.08
RPS6KA5	2.07
SLCO3A1	2.07
TNS3	2.07
IL17RC	2.05
LARGE	2.05
THBS1	2.05

PARP14	2.04
RBM47	2.04
C1ORF106	2.03
GM2A	2.03
CSPG4	2.03
SRP54	2.03
CCDC136	2.01
DBP	2.01
FLJ20273	2.00
C6ORF223	1.99
LOC440731	1.98
VASN	1.98
MAPRE3	1.97
C9ORF169	1.97
CGNL1	1.97
TSPAN1	1.97
FZD9	1.97
DHRS2	1.96
METTL7B	1.96
ANGPTL4	1.96
TAP1	1.95
EIF2AK2	1.95
ECGF1	1.95
CHSY3	1.95
ZNF467	1.95
MED25	1.94
LOC100131139	1.94
FTHL16	1.94
CNTNAP2	1.93
COL6A1	1.92
FTH1	1.92
PARP12	1.92
HMOX2	1.92
EPN3	1.92
XAF1	1.91

UPK1A	1.91
HS.434957	1.91
MEGF6	1.90
ZNFX1	1.90
KIAA1881	1.90
HLA-DMB	1.90
HERC5	1.90
HLA-C	1.90
KRT18P17	1.89
SLC16A5	1.89
C9ORF150	1.89
TRIOBP	1.88
GPR37	1.88
HPCAL4	1.87
KCNK3	1.87
OLFM1	1.87
LOC647081	1.87
EFHD2	1.87
FXYD3	1.87
TBC1D8B	1.86
GIYD1	1.85
SAMD9	1.85
LOC401321	1.85
EGLN1	1.85
ABCC2	1.85
SHROOM3	1.84
PGCP	1.84
LGALS3BP	1.84
TRIM5	1.84
PLCG2	1.84
HLA-H	1.83
TUBA4A	1.83
GDF5OS	1.83
SNORA6	1.83
MIR221	1.83

CAPN5	1.83
LIMCH1	1.82
TAP2	1.82
MAPRE2	1.82
TYMP	1.82
C19ORF66	1.81
UBE2H	1.81
SYN2	1.81
LOC100129781	1.80
RET	1.80
SLC25A22	1.79
PIR	1.79
IRF9	1.79
KCNS3	1.79
ZBED5	1.79
DOCK5	1.79
FBXO2	1.79
ASAP3	1.78
MLEC	1.78
C1ORF116	1.78
ISG15	1.77
PTK7	1.77
LOC345041	1.77
PHF11	1.77
MYO6	1.76
ITGA5	1.76
SNORA42	1.76
GCAT	1.76
CTSC	1.75
ASAP1	1.75
FBXW2	1.75
SRPX	1.75
PPP4R4	1.75
CHMP5	1.74
VIPR1	1.74

LOC388588	1.74
LOC646463	1.74
C4ORF34	1.74
PCSK5	1.73
FTL	1.73
HTATIP2	1.73
WBSCR27	1.73
PPFIBP2	1.73
SNORA1	1.73
DNASE1L1	1.73
GRN	1.72
RRAS	1.72
HS.105791	1.72
ST6GALNAC3	1.72
SKAP2	1.71
INPP4A	1.71
NMT2	1.71
GCDH	1.71
TDRD7	1.71
FAT1	1.70
PGM2L1	1.70
DSP	1.70
KIF5C	1.70
BCL3	1.70
PHLDB2	1.70
OMA1	1.69
PAM	1.69
RBPMS	1.69
CASP7	1.69
BAD	1.69
MVP	1.69
PCTK3	1.69
MSLN	1.69
ALDH1A3	1.69
CLDN23	1.69

TBC1D10A	1.68
ALDH6A1	1.68
NSF	1.68
PDE4A	1.67
MINPP1	1.67
C14ORF4	1.67
TIMP1	1.67
ACSL1	1.67
HRIHFB2122	1.67
FAM174A	1.67
SLC45A4	1.67
PROCR	1.66
CXCL16	1.66
CYP1B1	1.66
FSTL1	1.66
HS.31532	1.65
ASCC1	1.65
LOC643431	1.65
PRKAG2	1.65
FTHL11	1.65
ITPR1	1.65
SAMD5	1.65
PCOLCE2	1.64
CADPS2	1.64
SQLE	1.64
MBNL2	1.64
PELI2	1.64
C5ORF62	1.64
PNPO	1.64
BOLA2	1.63
LOC729009	1.63
ELF3	1.63
HS.356079	1.62
KRT80	1.62
ZFP36	1.62

KIAA0251	1.62
LHPP	1.62
LOC92249	1.62
PPP1R14C	1.62
HLA-G	1.62
CD24	1.62
NTN4	1.61
CHP	1.61
ALDOA	1.61
CLDND2	1.61
GCLM	1.61
CYP2U1	1.61
LOC283788	1.61
LOC389386	1.61
TSPAN3	1.61
MT1X	1.61
PRAF2	1.60
LRP8	1.60
UBE2G1	1.60
LRRN2	1.60
ID2	1.60
STOX1	1.60
SENP5	1.60
RDM1	1.60
NCRNA00081	1.60
PSME1	1.60
MSX1	1.59
TAX1BP1	1.59
GAD1	1.59
BCKDHB	1.59
CHPF	1.59
LRP10	1.59
PTPRE	1.59
HS6ST2	1.59
DOK4	1.59

GPC4	1.59
NKAP	1.58
F2RL1	1.58
ISG20	1.58
JAK1	1.58
ST6GAL1	1.58
42068	1.58
HSPA1B	1.58
PLS3	1.58
DYNC2H1	1.58
SHISA5	1.57
STEAP2	1.57
HLA-E	1.57
GCA	1.57
MGC11082	1.57
SORD	1.57
LOC402644	1.57
LOC643272	1.57
HPS3	1.57
BMP7	1.57
AGTRAP	1.57
OR51B5	1.57
FTSJD2	1.57
GSTP1	1.56
LOC642567	1.56
HLA-A29.1	1.56
LOC652595	1.56
ETNK1	1.56
PIK3CB	1.56
SEC24D	1.56
MALL	1.56
DUT	1.56
TRIM38	1.55
INSIG1	1.55
HSPH1	1.55

IFI27L2	1.55
LOC100129201	1.55
MICALCL	1.55
NOD2	1.55
B4GALT5	1.55
NUDT7	1.55
SCPEP1	1.55
DDR1	1.54
NFE2L3	1.54
HS.489254	1.54
FTHL3	1.54
TMEM151A	1.54
COMMD10	1.54
GPR180	1.53
TMC6	1.53
MTM1	1.53
TRIM21	1.53
MID1IP1	1.53
TPST2	1.53
PRDX3	1.53
F11R	1.53
CLDN7	1.53
CORO2A	1.52
CREG1	1.52
PEX6	1.52
FUT1	1.52
LEMD1	1.52
PRKAG1	1.52
SULT1A3	1.52
SCD5	1.52
TXNRD1	1.51
C2ORF18	1.51
CTSA	1.51
GRIN1	1.51
ESRRAP2	1.51

SEC24A	1.51
RAG1AP1	1.51
SLC39A11	1.51
B2M	1.50
HS.10862	1.50
GNG7	1.50
LAMP2	1.50
TMEM158	0.67
RACGAP1	0.67
DNAJC30	0.67
PPIL1	0.67
ZMAT3	0.66
UPF3B	0.66
WDR33	0.66
C8ORF45	0.66
POLR2J	0.66
RBM39	0.66
C1ORF19	0.66
RNF216	0.66
SNORA64	0.66
LOC100190986	0.66
SSTR2	0.66
RINL	0.66
SLC35F2	0.66
MND1	0.66
NLRP8	0.66
C14ORF85	0.66
DDX51	0.66
TARDBP	0.66
TIAL1	0.66
ZNF14	0.66
USP49	0.66
FAM127B	0.66
GOLGA1	0.66
ZNF738	0.66

IPO5	0.66
PKNOX1	0.65
SH2D3A	0.65
USF2	0.65
TNPO1	0.65
TUBB2A	0.65
TPM4	0.65
LOC388796	0.65
RN7SK	0.65
MGA	0.65
CIAPIN1	0.65
LRAP	0.65
PYGB	0.65
STC2	0.65
ODC1	0.65
EFCAB4A	0.65
CDKN2AIPNL	0.65
EIF2C2	0.65
SHARPIN	0.65
HS.193406	0.65
HNRPA1P4	0.65
SUPT4H1	0.65
BLZF1	0.65
RRAGB	0.65
MARK1	0.64
TUBB2C	0.64
RBM12B	0.64
TAF7	0.64
MYL6B	0.64
HS.158923	0.64
STAU2	0.64
C2ORF7	0.64
HS.143018	0.64
HELZ	0.64
CSNK1E	0.64

SIGIRR	0.64
MKKS	0.64
ZNF160	0.63
ZNF827	0.63
HNRNPU	0.63
HS.284464	0.63
MYBL1	0.63
THOC1	0.63
ZADH2	0.63
C22ORF32	0.63
CCBE1	0.63
EOMES	0.63
UGCGL1	0.63
LOC100129269	0.63
LOC650909	0.63
ANKRD11	0.63
LOC441124	0.63
DEM1	0.63
NAT10	0.63
MAST2	0.62
CDCP1	0.62
FLJ37644	0.62
LOC732360	0.62
WSB1	0.62
HBEGF	0.62
DENND1A	0.62
MBTD1	0.62
SNORD48	0.62
RASSF1	0.62
ZNF544	0.62
HCG2P7	0.62
LOC728903	0.62
LOC642934	0.62
HAUS8	0.62
LOC339352	0.62

CEP152	0.62
TSC22D3	0.61
TUBB4Q	0.61
DCBLD1	0.61
HS.558072	0.61
HS.561493	0.61
IL18BP	0.61
YTHDC1	0.61
AP3B1	0.61
LOC100129211	0.61
TNFAIP8L1	0.61
LOC153561	0.61
ZNF416	0.61
LOC100133795	0.61
HS.473191	0.61
ZNF483	0.61
WDR82	0.61
CCDC149	0.61
RC3H2	0.60
TACC3	0.60
STAG3L3	0.60
MPZL1	0.60
FOXD4L1	0.60
KIAA1751	0.60
NASP	0.60
CHRNA5	0.60
LOC644695	0.60
LOC100128098	0.60
RPL28	0.60
LOC730092	0.60
CHRNA10	0.60
LOC729423	0.60
PHC2	0.60
UPF3A	0.60
C20ORF94	0.60

HS.516646	0.59
HS.574671	0.59
ZNF83	0.59
HRAS	0.59
PLK2	0.59
TAF15	0.59
HS.171171	0.59
HNRNPA1	0.59
XRCC2	0.59
LOC728653	0.59
TMEM17	0.59
PRUNE	0.59
RAPGEF6	0.59
LOC100128440	0.59
RPS23	0.59
CHD4	0.58
CDKN2B	0.58
LOC100132585	0.58
MAMDC4	0.58
CCDC106	0.58
OTUB1	0.58
UBN2	0.58
MAP1LC3B	0.58
LOC389765	0.57
SCRIB	0.57
CPVL	0.57
TMEM44	0.57
ATP5D	0.57
ZNF789	0.57
LAMB1	0.57
ZNF37A	0.57
MSL3L1	0.57
CRYZL1	0.57
LOC728452	0.57
PRR11	0.57

FYTTD1	0.57
MARVELD3	0.57
GALNT3	0.56
GTSE1	0.56
ZNF562	0.56
ZFP90	0.56
HNRPA1L-2	0.56
FAM107B	0.56
LOC113386	0.56
AMT	0.56
FOXC1	0.56
LOC649841	0.56
CDKN2A	0.56
RNY1	0.55
FOXD1	0.55
EFNB2	0.55
HS.62314	0.55
TRIM13	0.55
TOP3A	0.55
YPEL1	0.55
RNY5	0.54
HS.561915	0.54
POLE	0.54
SYNE2	0.54
DLX1	0.54
FKBP2	0.54
TMEM86B	0.54
GLS	0.53
LETM2	0.53
HIST1H4K	0.53
WNT7B	0.53
PKMYT1	0.53
ATXN7L2	0.53
ZNF84	0.53
LOC100133923	0.53

DDX39	0.53
HS.576072	0.52
ZNF525	0.52
LOC653524	0.52
ZNF480	0.52
RECQL4	0.52
METT11D1	0.52
RPL37A	0.52
SDHALP1	0.52
LOC100131578	0.52
NUDT1	0.52
JAG1	0.51
YJEFN3	0.51
FBXO32	0.50
LOC283932	0.50
CRYGS	0.50
HEY1	0.50
LEP	0.49
TMOD2	0.49
ZNF500	0.49
CLEC11A	0.49
NGDN	0.48
C6ORF26	0.48
WDFY3	0.48
SF1	0.48
GJC2	0.48
ZNF12	0.48
CBX5	0.48
PHF20L1	0.48
AMH	0.48
MACF1	0.48
NPR2	0.48
VPS13B	0.47
SPATA13	0.46
MRP63	0.45

LOC642678	0.45
MAMDC2	0.45
SPIN1	0.43
HS.408455	0.43
HS.184721	0.43
C1ORF104	0.43
TRIM36	0.42
FLJ23754	0.42
HS.145444	0.42
AP1S2	0.40
HS.548302	0.40
HS.157344	0.40
BMP4	0.40
C16ORF79	0.40
TXNDC12	0.39
42069	0.39
COCH	0.38
LOC100170939	0.37
KCNH8	0.37
OPA3	0.37
GLIS3	0.36
GAS6	0.36
LOC653352	0.35
C7ORF54	0.35
LOC100129148	0.34
OVOL2	0.34
ZNF91	0.33
GPT	0.33
SRPX2	0.33
SLC38A5	0.32
FES	0.32
LIN7B	0.32
NANOS1	0.32
C2ORF67	0.32
LOC100132761	0.32

SYNJ2	0.32
IFNE1	0.31
ONECUT2	0.30
C19ORF30	0.30
LOC388969	0.29
HS.570821	0.29
MGC16384	0.29
HS.577098	0.28
SCG2	0.26
KLHL13	0.26
PRF1	0.25
HS.444999	0.25
ATXN2L	0.23
ZNF608	0.14
ESYT1	0.12
IGFL1	0.11
LOC401074	0.09
GPM6B	0.08
LOC441081	0.06
FGF9	0.06
NRIP1	0.04
MUC4	0.03
ZNF711	0.02
ZNF493	0.01

Table S1: Genes differentially expressed in P1 as compared to O1 and O2 (fold-change ≥ 1.5 and FDR ≤ 5%). P1; polymetastatic clone, O1 and O2; oligometastatic clones.

Suupplementary Table S2

Gene Symbol	Fold-Change (P2 vs. O)
MB	20.16747627
HS.484967	17.2063854
ATPGD1	10.65290534
SUNC1	10.18005652
SPHK1	7.757852656
UBC	7.21385902
HECW2	6.188679245
TTN	6.150259067
HLA-DMB	6.074761255
FAM83A	5.824046921
RHBDL1	5.79046853
MYH15	5.45622621
SPRR2F	5.226238532
MT1G	4.734536082
EID3	4.519258203
CLGN	4.300736067
POTEE	4.284671533
PDE2A	4.204692737
SPINK1	4.138344227
SPRR2D	4.077386964
PLAU	3.995041509
STK32A	3.986607143
MTE	3.645338517
RN7SK	3.5029036
C6ORF59	3.475096178
LOC441019	3.437524079
C20ORF127	3.425492561
IGFL3	3.371474617
LOC644100	3.316280384
IL1A	3.192192192
CLEC4D	3.160409556
UPP1	3.139298705
KCNH3	3.004459309

ABCB1	2.975824176
COL6A3	2.959752322
C11ORF68	2.95607362
DIRAS3	2.951248514
RBBP6	2.950391645
MT1X	2.890934673
PAPSS2	2.84885127
SNORA79	2.788515406
SCG2	2.712051734
MALL	2.702924417
LOC644655	2.659638554
GPNMB	2.647540984
LOC338758	2.635687732
HS.569566	2.610259122
LOC729231	2.604620892
EVI1	2.599727149
SERPINA5	2.592736706
MT1A	2.570655336
RBCK1	2.563842885
FAM110C	2.5625
EDNRA	2.550033135
AGPAT4	2.539649846
MT1E	2.530733378
AURKAPS1	2.516276413
BAMBI	2.515147205
ERN1	2.458373206
MT2A	2.408542394
LOC283487	2.38778626
LOC100128163	2.384989374
C18ORF19	2.353176089
LOC647349	2.32942064
ATG12	2.317178274
GEM	2.310252281
GAS6	2.292728821
DNAJC12	2.27536866

LIX1L	2.268991283
LOC100130009	2.268737798
P8	2.266137041
PDLIM3	2.262869927
LOC143188	2.256880734
OR2A42	2.253882512
PPP1R1C	2.246464646
MUC4	2.23964497
ANKRD29	2.237625802
FOSL1	2.225594949
NT5E	2.214512209
CXCL1	2.206680585
SAMD5	2.198138298
PRPH	2.192202144
HS.291319	2.177954847
ASPH	2.141585184
LAT2	2.108534684
SCARNA8	2.087635575
BCL2	2.080303852
LRAT	2.078720787
AP3S1	2.075465609
KSR2	2.059805285
SCARNA14	2.049816466
RNF144B	2.041970803
OR2A20P	2.035966602
TRIM10	2.032040472
HLA-DMA	2.004476701
HS.493947	1.984682713
EHBP1	1.984394649
BAX	1.984256157
GJB4	1.971372804
C14ORF72	1.968126691
HS.568777	1.9625
NUPR1	1.945304066
KLK5	1.937806296

RBPJ	1.91681736
FHL1	1.915978995
TOX2	1.91276252
INPP4A	1.910469314
ACBD7	1.908675799
OR2A9P	1.908288281
LOC221710	1.903067485
ACSM3	1.886339318
CCDC85B	1.879183673
PDGFC	1.872867864
HMGA1	1.871164706
SLC7A11	1.862153095
CPLX1	1.854241553
PLEKHA9	1.849975526
FLYWCH1	1.847828282
TSC22D1	1.844646331
STAMBPL1	1.838942009
FTHL16	1.831115842
HS.553301	1.825706791
CXORF57	1.825368841
ZFP36	1.823666803
LOC643272	1.816884595
BEND6	1.803278689
COBLL1	1.799986177
PIP5K2A	1.798143852
GPR177	1.794612019
TMEM17	1.787896254
TROVE2	1.785780813
LOC100128269	1.782218597
LAMC2	1.781131479
KRT222	1.776975821
LOC642732	1.776888889
LOC649604	1.775820657
PARP8	1.765798526
FTHL3	1.763219944

SEPHS2	1.760058962
LAMC1	1.742268861
LOC100133220	1.739191074
STXBP5	1.737150586
LTBR	1.7353909
LOC642567	1.734850914
UNC5B	1.734223562
PITPNC1	1.728482373
KLF4	1.725716826
CTAGE6	1.721334809
NUP98	1.719708862
FTHL11	1.716897167
LOC441294	1.71334522
GUCA1B	1.703660662
HS.575603	1.700091158
CCDC68	1.699902439
DNAJC22	1.697785835
LOC100133673	1.695550351
LOC729009	1.693813781
SCML2	1.684089162
ASNS	1.679464438
TK2	1.678740157
HS.568329	1.674410293
TOP1P2	1.672519026
LOC644936	1.670935034
PPIL6	1.667692308
LOC729952	1.666806899
BRAF	1.665742574
AXL	1.662797927
LARP6	1.661581365
FTHL2	1.661206961
RAB38	1.660531915
SNORA11D	1.657784744
F11R	1.65720524
DNAJC10	1.652206714

CHAC1	1.651372697
UBE2E1	1.648288288
LOC646476	1.645201238
TNFRSF6B	1.644192506
SESN2	1.642809064
HS.372654	1.642587845
PYGB	1.639057342
DNMT3B	1.637205387
PDLIM7	1.637025481
CD55	1.63690369
LOC399491	1.6366171
GART	1.635081899
CTSL1	1.629623974
C9ORF150	1.628930818
CENTB2	1.625695054
FAM167A	1.62435785
RINL	1.622109559
MERTK	1.619107877
STOX1	1.619098426
MOBKL2A	1.614913958
LOC147727	1.612751893
KBTBD9	1.612205599
FKSG30	1.609084345
C9ORF72	1.608290778
ELK4	1.591985428
HS.27048	1.591978035
42066	1.58968059
MSRA	1.589160192
TOP1	1.587122527
STK17B	1.586073501
CR2	1.581733566
C2ORF37	1.573072497
TSEN15	1.572467625
LOC652900	1.571981092
TXNRD1	1.570924135

MEGF6	1.570629954
FAM72A	1.569230769
DPY19L1	1.566139165
KLHL29	1.566006224
SLC39A3	1.565704581
FTHL8	1.563999501
KIAA1881	1.55704698
CLIC4	1.55525787
DNAL1	1.554137664
LOC388692	1.552025932
PTPRH	1.547845551
RHOG	1.545325328
DLEU1	1.545276661
DNAJB14	1.541769475
NQO2	1.537566419
BTG1	1.537452415
CDC25B	1.536586554
LOC440928	1.534389882
PREPL	1.533585013
PDIA3P	1.531882971
TMLHE	1.531075868
MYBL1	1.529695799
SH3BGRL2	1.529370507
IFFO1	1.527410372
HMGN2	1.526611186
MRPS10	1.525603445
WRB	1.525091799
TXNDC12	1.523259041
PPP3R1	1.523155441
TMEM136	1.522656578
C11ORF73	1.521968366
RFC5	1.521697203
RHOF	1.517058042
BEX2	1.515544466
RNMT	1.514644559

GTPBP8	1.51456068
GNL3	1.514177987
LOC728855	1.510473095
HS.544451	1.509191176
LOC392437	1.506467811
C12ORF48	1.50637903
TUBG1	1.504388057
COQ10A	1.501902174
GALNS	1.501358942
FOXC2	1.501274117
DAPK3	0.666462293
MIR221	0.665314539
KLHL24	0.664870024
PCTK3	0.663416846
EGLN1	0.663070006
OSBPL7	0.662597114
EFNA1	0.661224174
RNF31	0.65927905
NUAK1	0.659109868
BMP1	0.658511555
LTBP3	0.655823893
PDCD6IP	0.655429924
FBXO2	0.653509214
CAB39	0.653246783
MAMDC2	0.651099436
HS.284464	0.651097454
SYT1	0.650895334
HS.154336	0.64853779
TMEM45A	0.648396138
PODXL	0.647356413
FAM131C	0.646779661
CNFN	0.645487173
PCBP4	0.644207631
PHLDA3	0.644181926
FGD3	0.642667311

PRR5	0.642075781
ABHD7	0.642002684
SMAD7	0.640887934
FDFT1	0.640532219
ZNF160	0.640392963
RAB11FIP3	0.640142518
SLC4A11	0.639568644
PCMTD1	0.63911907
HRASLS3	0.63857356
SULT1A4	0.6379964
MAPKAP1	0.637760328
ITPR3	0.637588643
STK36	0.637127164
CHKA	0.63700576
FAT1	0.636374102
HNRPDL	0.635391311
N4BP1	0.634832329
PDXK	0.633461481
HMG20B	0.632875748
ZNF320	0.632303181
C12ORF4	0.631284377
NEURL4	0.62999797
TAX1BP3	0.627936205
EIF3K	0.627907496
HS.499716	0.62771503
HERC5	0.626855665
HLA-F	0.625894378
PIK3CD	0.625060946
LOC730417	0.624382716
FSTL3	0.62417772
NOV	0.623487544
SNW1	0.622642309
DNAJB2	0.621523359
GAD1	0.621515892
INPP4B	0.619374523

KRT19	0.615725364
TMC6	0.615430179
CXCL16	0.614966976
SP100	0.613674892
SLC27A1	0.613205117
LOC728229	0.613017751
NDRG2	0.609484536
INF2	0.608593092
TRPM4	0.607551127
TP53INP1	0.606768467
LY6E	0.605549046
SYTL1	0.605008078
PRRX2	0.602918379
TRIM33	0.602195504
PPAPDC1B	0.60126998
CRIP2	0.60113387
FUCA1	0.599436257
GPC2	0.59789644
CASP6	0.596033403
NOXA1	0.595757637
CD151	0.595034835
HBE1	0.594725873
SLC2A6	0.594053109
IFNAR2	0.593673219
PARP14	0.589425542
ARL6IP5	0.588922177
GLB1L2	0.588661552
SCAPER	0.588235294
GARNL3	0.584699454
GPR137B	0.582453586
TMEM132A	0.580179462
TAS2R10	0.579302587
PAN2	0.577662722
ZNF322A	0.577532228
MGC4677	0.573676041

LOC100134134	0.5721677
SHROOM3	0.571626997
C11ORF80	0.570589737
ANKDD1A	0.570070708
ZNF177	0.569397993
GFI1	0.568965517
HS.434957	0.565596081
WNT3A	0.563248308
HS.157344	0.561238596
KCNQ2	0.559961114
TGFBR2	0.55795807
IKBKB	0.553538348
IFIH1	0.553329343
CBX7	0.552660362
SULT2B1	0.551964512
COL13A1	0.551773558
HS.184721	0.548826382
TMEM191B	0.547988965
LOC647954	0.544764795
ARHGEF17	0.544093178
HS.91389	0.544015735
C17ORF28	0.540871058
PHLDA1	0.54076059
NTN4	0.540302613
CTDSPL	0.539996136
CDK5RAP3	0.538287307
FGFR3	0.537694456
ID2	0.537642151
MAMDC4	0.533423975
HS.568928	0.529179959
HS.19339	0.52794755
SPG7	0.52688172
CACNB3	0.526628112
CD109	0.52441009
LRRN2	0.518729844

TMOD2	0.51546894
MICAL1	0.512716175
SOX8	0.505881827
CHD5	0.504714863
STAT1	0.501068851
SLC36A1	0.500991408
LOC100128440	0.5
HS.202140	0.499298344
CUEDC1	0.498301409
TCIRG1	0.497766951
ALDH1A3	0.495319161
EMP1	0.492485213
ACTA2	0.490685205
GDPD5	0.490020143
FBN3	0.481557377
SLCO3A1	0.475465957
SP110	0.469827586
PTPRE	0.467730875
C5ORF62	0.464106845
ARSG	0.463399243
MEIS3	0.459128065
TMEM159	0.457227395
SRPX2	0.447639302
TNFSF9	0.441480381
HERC6	0.429858282
C9ORF61	0.422545726
PDLIM1	0.421795732
C1ORF116	0.420876771
ZNF322B	0.41634189
CCL5	0.414173492
PLXNB1	0.411174785
MMP11	0.408341232
PLK2	0.408230294
FGFBP1	0.405732223
SULF2	0.403258656

REC8	0.40164323
SEMA3B	0.397231365
BMP4	0.396981722
FLJ39632	0.392914654
IRF7	0.390203056
PLA2G10	0.388850936
42250	0.378027403
ATOX1	0.376444629
ISM1	0.371503825
TNNC1	0.368214717
APOBEC3F	0.364279399
MGC26718	0.356642161
ADAMTS14	0.345803207
BCORL1	0.338076546
DUOX1	0.328331894
IRF9	0.326909108
ALS2CL	0.320845341
ISG15	0.318315876
C21ORF81	0.31785848
KCNH8	0.302934179
SPIN1	0.296932515
S100A2	0.295107942
CSF2	0.294416244
HS.538535	0.288855572
APOBEC3C	0.283721453
DDX60	0.273894975
PMEPA1	0.269424577
KRT80	0.267845717
HS.13291	0.25320787
FLJ46380	0.250704225
COL17A1	0.244490653
IFIT2	0.230348235
OASL	0.22995408
GLIS3	0.201069996
GSTA4	0.191721133

EPSTI1	0.166109626
PRF1	0.103817543
RHOT2	0.084711166
IFI27	0.050071887
CLIC5	0.037842952
GLI2	0.033898305
IGFL1	0.011630322
LARGE	0.000762631

Table S2: Genes differentially expressed in P2 as compared to O1 and O2 (fold-change ≥ 1.5 and FDR ≤ 5%). P2; polymetastatic clone, O1 and O2; oligometastatic clones.
